# Renal CD81 interacts with sodium potassium 2 chloride cotransporter and sodium chloride cotransporter in rats with lipopolysaccharide‐induced preeclampsia

**DOI:** 10.1096/fj.202201546RR

**Published:** 2023-03-24

**Authors:** Ping Wang, Gangyi Zhu, Qiaozhen Wu, Li Shen, Dan Liu, Zhiyin Wang, Weiwan Wang, Zhiyun Ren, Yutao Jia, Mingda Liu, Ying Xue, Daxi Ji, Yali Hu, Yanting Yu, Xiaoyan Wang

**Affiliations:** ^1^ The Core Laboratory for Clinical Research, BenQ Medical Center The Affiliated BenQ Hospital of Nanjing Medical University Nanjing China; ^2^ Department of Nephrology, BenQ Medical Center The Affiliated BenQ Hospital of Nanjing Medical University Nanjing China; ^3^ Department of Obstetrics and Gynecology, BenQ Medical Center The Affiliated BenQ Hospital of Nanjing Medical University Nanjing China; ^4^ Department of Obstetrics and Gynecology, Nanjing Drum Tower Hospital Nanjing University Medical School Nanjing China

**Keywords:** mouse renal DCT cells, NCC, NKCC2, patients, rat preeclampsia model, renal CD81

## Abstract

The kidney regulates blood pressure through salt/water reabsorption affected by tubular sodium transporters. Expanding our prior research on placental cluster of differentiation 81 (CD81), this study explores the interaction of renal CD81 with sodium transporters in preeclampsia (PE). Effects of renal CD81 with sodium transporters were determined in lipopolysaccharide (LPS)‐induced PE rats and immortalized mouse renal distal convoluted tubule cells. Urinary exosomal CD81, sodium potassium 2 chloride cotransporter (NKCC2), and sodium chloride cotransporter (NCC) were measured in PE patients. LPS‐PE rats had hypertension from gestational days (GD) 6 to 18 and proteinuria from GD9 to GD18. Urinary CD81 in both groups tented to rise during pregnancy. Renal CD81, not sodium transporters, was higher in LPS‐PE than controls on GD14. On GD18, LPS‐PE rats exhibited higher CD81 in kidneys and urine exosomes, higher renal total and phosphorylated renal NKCC2 and NCC with elevated mRNAs, and lower ubiquitinated NCC than controls. CD81 was co‐immunoprecipitated with NKCC2 or NCC in kidney homogenates and co‐immunostained with NKCC2 or NCC in apical membranes of renal tubules. In plasma membrane fractions, LPS‐PE rats had greater amounts of CD81, NKCC2, and NCC than controls with enhanced co‐immunoprecipitations of CD81 with NKCC2 or NCC. In renal distal convoluted tubule cells, silencing CD81 with siRNA inhibited NCC and prevented LPS‐induced NCC elevation. Further, PE patients had higher CD81 in original urines, urine exosomes and higher NKCC2 and NCC in urine exosomes than controls. Thus, the upregulation of renal CD81 on NKCC2 and NCC may contribute to the sustained hypertension observed in LPS‐PE model. Urine CD81 with NKCC2 and NCC may be used as biomarkers for PE.

## Graphical abstract

CD81 interacts with NKCC2 and NCC in LPS‐preeclampsia rat kidneys and positively regulates NCC in cultured mouse renal‐distal‐convoluted‐tubule cells. The enhanced recruit of NKCC2 and NCC into renal plasma‐membranes in LPS‐preeclampsia rats links to upregulations of CD81. Those changes may contribute to the sustained‐hypertension observed in LPS‐preeclampsia model. Higher CD81 with NKCC2 and NCC in preeclampsia patients than controls are seen in original urines and urine exosomes, and may be used as noninvasive biomarkers for preeclampsia.

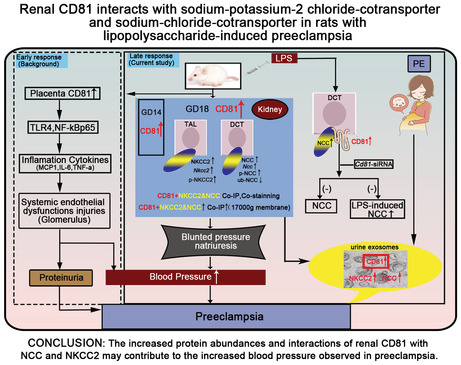

**Abbreviations**
α‐NKA: sodium‐potassium ATPase‐α
AEC: angiotensin converting enzyme
AT1: type 1 angiotensin II receptor
BP: blood pressure
CCK‐8: cell counting kit 8
CD81: cluster of differentiation 81
Co‐IP: co‐immunoprecipitation
CTL: controls
DAPI: 4,6‐diamino‐2‐phenyl indole
DCT: distal convoluted tubules
DTT: dithiotrietol
ECL: enhanced chemiluminescence
ENaC: epithelia sodium channel
GD: gestational‐days
H&E: haemotoxylin and eosin
HRP: horseradish peroxidase
IL‐6: interleukin 6
IM: intracellular‐vesicle‐enriched membrane fractions
LDH: lactate dehydrogenase
LPS: lipopolysaccharide
MCP1: monocyte chemoattractant protein‐1
mDCT: mouse renal distal‐convoluted‐tubule
NaPi2: type 2 sodium phosphate cotransporter
NCC: sodium‐chloride‐cotransporter
NEDD4L: neural precursor cell expressed, developmentally down‐regulated 4‐like
NHE3: type 3 sodium hydrogen exchanger
NKCC2: sodium‐potassium‐2 chloride‐cotransporter
PE: preeclampsia
PM: plasma‐membrane‐enriched fractions
pNKCC2: phosphorylated NKCC2
Q‐PCR: quantitative real‐time PCR
RAAS: renin‐angiotensin‐aldosterone system
SBP: systolic blood pressures
TAL: thick ascending limbs
TCL: total cell lysates
TNF‐α: tumor necrosis factor‐α
WKH: whole kidney homogenates
WNK4: with no lysine kinase 4

## 1 INTRODUCTION

Preeclampsia (PE) is a pregnancy‐specific syndrome affecting 5%–7% of pregnancies globally and is the leading cause of pregnancy‐related maternal and perinatal mortality and morbidity, especially in low‐ and middle‐income countries.^1^, ^2^ The primary symptom of PE is hypertension. However, the exact etiology of the syndrome remains unknown. Our study explores the potential role of the cluster of differentiation 81 (CD81), a transmembrane 4 superfamily protein, in the pathogenesis of hypertension in PE.^3^

The kidney is the most important organ for the chronic regulation of blood pressure (BP)^4^ primarily through its effects on salt and water reabsorption by renal tubules.^5^, ^6^ The homeostasis of salt and water largely depends on the expression and function of the sodium transporters, channels, and pump in the renal tubules.^7^ Major apical sodium transport proteins include type 3 sodium hydrogen exchanger (NHE3) and type 2 sodium phosphate cotransporter (NaPi2) in proximal tubules, sodium potassium 2 chloride cotransporter (NKCC2), and NHE3 in thick ascending limbs (TAL) of the loop of Henle, sodium chloride cotransporter (NCC) in distal convoluted tubules (DCT), and epithelial sodium channel (ENaC), which has 3 subunits (α, β, γ), in the later DCTs, connecting tubules and collecting ducts. Sodium potassium ATPase‐α (α‐NKA) is located in the basolateral membrane of nearly all segments in the renal tubules.^8^, ^9^ The suppression of these sodium transport proteins is triggered by increases in renal perfusion pressure and results in an increase of sodium excretion with a negative balance in the extracellular fluid volume.^10^ This physiological phenomenon, known as pressure natriuresis, is a key mechanism in BP regulation by kidney.^8^, ^11^ A marked decrease in NCC in renal DCTs is found in a rat model with aldosterone escape, a manifestation of the pressure natriuresis phenomenon.^8^ However, the role of renal tubular sodium transport proteins in BP regulation during pregnancy is not well‐understood.

Renin–angiotensin–aldosterone system (RAAS) is known to play an important role in renal salt and water reabsorption and BP regulation^12^, ^13^, ^14^ and in pregnancy‐related hypertension.^15^ However, studies have shown that RAAS‐independent factors also regulate renal sodium reabsorption by affecting transport proteins.^9^, ^11^, ^16^, ^17^, ^18^ Increases in NKCC2, NCC, and ENaCs have been observed in the urinary extracellular vesicles from patients with PE.^19^, ^20^ This suggests that uninhibited or increased sodium transporter proteins in renal tubules may contribute to the development of hypertension during pregnancy. To date, research has not identified a clear mediator of these increases in urinary sodium transporters in the development of hypertension in PE.

CD81 is a widely expressed tetraspanin that provides a scaffold for signaling molecules and orchestrate interactions between membrane‐associated proteins.^21^, ^22^, ^23^ CD81 is also a main component of exosomes (a major format of extracellular vesicles) in various cell types.^24^, ^25^ Prior research has observed elevated CD81 in the placenta and sera of PE patients and PE models,^26^ but there has been no study into whether endogenous CD81 in the kidney plays a role in the regulation of the tubular membrane proteins associated with sodium reabsorption during pregnancy. Researchers have observed CD81, together with hundreds of PE‐unique proteins, in the syncytiotrophoblast microvesicles of perfused PE placentas through multi‐dimensional protein identification technology analysis.^27^ In addition to the placenta, an increase in CD81 has also been observed in the sera, serum exosome‐free fractions, and serum exosomes from patients with PE.^26^, ^28^ Over‐expression of CD81 by transfecting CD81‐adenovirus into pregnant rats on gestational day (GD) 5 mimics the phenotype of PE, including hypertension and proteinuria; CD81‐oriented vascular dysregulation has been linked to the interruption of endothelial functions in primary‐cultured human umbilical vein endothelial cells and in the reduction of the artery diameters in uterine mesomentrial triangle found in CD81‐induced PE rat models.^26^

In this study, we tested the hypothesis that interactions of renal CD81 with sodium transport proteins contribute to the pathogenesis of hypertension in PE. We utilized a general PE model by infusing a small amount of lipopolysaccharide (LPS) into the tail veins of pregnant rats on GD5,^29^ in which the hypertension occurs on GD6 to GD18 and proteinuria appears on GD9 to GD18. Renal CD81, sodium transporters, RAAS components, and pro‐inflammatory cytokines were studied on GD14 and GD18 in LPS‐PE rats. The interactions between CD81 and sodium transporters were determined in rat kidneys and cultured renal distal convoluted tubule cells treated with *cd81*‐siRNA in the presence or absence of LPS stimulation. Their associations in different membrane fractions were explored in the kidneys from LPS‐PE model. The protein abundances of CD81, NKCC2, and NCC were also evaluated in original urines and urinary exosomes from PE patients relative to healthy pregnant women.

## 2 MATERIALS AND METHODS

### 2.1 Pregnant rats treated with LPS or vehicle

Sprague–Dawley rats (10 weeks old) were purchased from the Animal Center of Nanjing Medical University and raised in a light‐ and humidity‐controlled room with free access to food and water. The female rats were rendered pregnant by being housed with fertile males at a 2:1 ratio overnight. The day when pregnancy was confirmed by the presence of vaginal spermatozoa was designated as a gestational day (GD) 0. The pregnant rats were divided (simple randomization) into LPS‐PE and vehicle groups and treated with a single ultra‐low dose of lipopolysaccharide (LPS, 0.5 μg/kg, dissolved in 2 mL of saline) or vehicle, respectively, through tail vein by an infusion pump (2 mL/h) on GD5 with restraining‐devices. BPs were measured by tail‐cuff method at 8–10 a.m. (BP‐98A, Softron, Tokyo, Japan) while urines were collected in metabolic cages (8 p.m.–10 a.m.) for protein measurement every 3 days^30^ during the pregnancy. After being euthanized by cervical dislocation under isoflurane‐anesthesia on GD18, the kidneys, placentas, livers, and sera were collected and frozen immediately for further analyses. In additional rats, the experiments were terminated on GD14 for studies on kidneys, as indicated. All procedures were performed under the guidelines of the Experimental Animals Management Committee (Jiangsu Province, China) and ethics approval by the Animal Care and Use Committee of Nanjing Drum Tower Hospital (SYXK 2009‐0017). The same rat‐PE models are reported previously,^29^, ^31^, ^32^ and the samples of kidney, placenta, liver, serum, and urine at GD18 were achieved from the experiments published recently.^32^

### 2.2 Preeclampsia (PE) patients and controls (CTL)

This study was approved by the Ethics Committee of BenQ Hospital, Nanjing Medical University (2021‐KL009). Written informed consent was obtained from all participants. The human subjects included patients with PE and normal pregnant women dated from January 2019 to November 2022. All women are pregnant with singleton pregnancies, and all pregnancies were naturally conceived. The untreated inpatients were diagnosed with PE according to the criteria^33^ of The American College of Obstetricians and Gynecologists, defined as new‐onset hypertension, after 20 weeks of gestation with systolic BP ≥140 mmHg, and/or diastolic BP ≥90 mmHg, accompanied by proteinuria (≥300 mg/24 h). The clinical data were collected at gestational weeks, when PE was diagnosed and urine samples were collected. Pregnant women with combined diseases and other gestational complications were excluded. The CTL group consisted of gestation week‐matched women from the antenatal care. BPs (seated, left or right upper arm at the same level as heart, with appropriate arm‐cuff) were measured twice at 4 h or more apart with automated devices (HBP‐1300; OMRON, Da Lian, China) by medical professionals. Spot urine specimens were collected and frozen immediately at −80°C until urine experiments were performed.

### 2.3 Mouse distal convoluted tubule (DCT) cells, LPS stimulation, and siRNA interference

Immortalized mDCT cells were gifts from Dr. Peter Freidman at the University of Pittsburgh.^30^ The purity of the DCT cells was verified by detecting positive NCC but negative NHE3, NKCC2, and αENaC by immunoblotting. The mycoplasma‐free cells (passages 35–37) were cultured in Dulbecco's Modified Eagle Medium (DMEM)‐F‐12 (11320033, Gibco) containing heat‐inactivated 10% fetal bovine serum (FBS500‐S, AusGeneX) and 10 μg/mL of antibiotic–antimycotic mixture (A5900, Sigma) at 37°C in an incubator with humidified 5% CO_2_ and 95% air (MCO‐15 AC, Sanyo). The cells were seeded and grew in multi‐well culture plates (70%–80% confluence), and then treated with LPS (L2880, Sigma)^34^ in serum‐free DMEM‐F‐12 or transfected using Lipofectamine RNAiMax (L3000‐015, Invitrogen) with small interfering RNA oligonucleotides^35^ of *Cd81*(*Cd81*‐siRNA: 5′‐3′GCACCAAAUGCAUCAAAUATT, 5′‐3′UAUUUGAUGCAUUUGGUGCTT) and mock (General Biosystems, China). The cell viability and toxicity were determined by using a cell counting kit 8 (CCK‐8) (C0042, Beyotime) for growth rates and using a diagnostic kit (C0017, Beyotime) for LDH release rates according to the manufacturer's protocol.^35^

### 2.4 Antibodies

The sources of antibodies were: mouse monoclonal antibodies against CD81(B‐11, SC‐166029, Santa Cruz, RRID:AB_2275892), ubiquitin (646302, Biolegend, RRID:AB_1659269), phosphorylated serine (05‐1000 X, Upstate, RRID:AB_1128624) and α1 NKA (05‐369, Sigma, RRID:AB_309699); rabbit polyclonal antibodies against actin (A2066, Sigma, RRID:AB_476693), GAPDH (60004‐1‐Ig, Proteintech, RRID:AB_2107436), WNK4 (with no lysine kinase 4, 5713, Cell Signaling, RRID:AB_10544699), NEDD4L, or NEDD4‐2 (13690‐1‐AP, Proteintech, RRID:AB_2149326), Phosphorylated threonine/tyrosine (9381, Cell Signaling, RRID:AB_330301), AT1 (type 1 angiotensin II receptor, N10, Santa Cruz, RRID:AB_2801404), ACE (angiotensin‐converting enzyme, 24743‐1‐AP, Proteintech, RRID:AB_2879701), ACE2 (21115‐1‐AP, Proteintech, RRID:AB_10732845), MCP‐1 (ab25124, Abcam, RRID:AB_448636), IL‐6 (ab214429, Abcam, RRID:AB_2889390) and TNF‐α (17590‐1‐AP, Proteintech, RRID:AB_2271853); the rabbit polyclonal antibodies against NHE3, NaPi2, NKCC2, pNKCC2 (phosphorylated NKCC2), NCC, ENaCs were gifts from Dr. Mark Knepper in NIH. The specificity of CD81 antibody is reported in CD81 over‐expression samples^26^ and also was characterized by detecting a linear correlation between target band intensities and CD81 peptide concentrations. Horseradish peroxidase (HRP)‐conjugated or fluorescent secondary antibodies were purchased from Abcam or Invitrogen.

### 2.5 Semi‐quantitative immunoblotting

The kidneys or cell suspensions were ultrasonicated on ice in Isolation Solution containing 10 mM triethanolamine (102‐71‐6, Macklin) and 250 mM sucrose with protease/phosphatase Mini Tablets (A32961, Pierce). Protein concentration was measured with BCA kit (P0009, Beyotime) and adjusted to 2 μg/μL before the samples were prepared in loading buffer (P0015L, Beyotime) and 0.75% dithiothreitol (DTT, D8220, Solarbio) and denatured at 60°C for 10 min. A Coomassie Brilliant Blue‐stained loading gel was used to equalize the protein concentration by averaging 3 bands, at upper, middle, and lower sizes, each sample. Serum samples, diluted at 1:7, and urine samples without dilution, were denatured in loading buffer with DTT. 10–40 μg of protein samples were separated by electrophoresis on SDS‐PAGE and transferred (BIO‐RAD system) onto nitrocellulose membranes (66458, Bio Trace), which were incubated in primary antibodies at 4°C overnight on a low‐speed shaker. HRP‐conjugated secondary antibodies plus chemiluminescence's kit (ECL, E412‐02, Vazyme) were used to visualize the target protein bands with Tanon 5200 Multi system. The band intensities were measured using ImageJ, corrected with intensities of corresponding actin or GAPDH and calculated as a percentage of controls.^8^, ^9^

### 2.6 Immunofluorescence with confocal microscopy

Paraffin‐embedded kidney sections (2 μm) were de‐waxed with xylene and rehydrated with ethanol sequentially (99%, 96%, 75%). The antigen retrieval was done by 15 min boiling in citrate repair solution (Ph 6.0), After blocking and washing, the sections were incubated with primary antibodies, then fluorescence‐conjugated secondary antibodies. In addition, methanol‐fixed cell slips were similarly incubated with antibodies. Nuclei were stained with DAPI on kidney sections or Hoechst on cell slips. The slides were mounted using ProLong Gold Antifade Mountant (Thermo Fisher Scientific) and imaged with LSM 800 confocal microscope (Carl Zeiss, Oberkochen, Germany).^8^, ^9^ Kidney structure was regularly stained with Haemotoxylin and Eosin (H&E) and viewed with Nikon Eclipse Ci‐E microscope. At least 4 views at 3, 6, 9, 12 o'clock positions were taken in each kidney section, *n* = 2–3/group.

### 2.7 Co‐immunoprecipitation

Uniform amounts (100 μg) of protein were immunoprecipitated (pull‐down) with 1 μg of first primary antibodies, such as NCC and NKCC2 (rabbit), using ProteinA+G beads (P2055, Beyotime). Normal rabbit (#14708, Cell Signaling Technology) or mouse (AB 2770414, ABclonal) IgG was used as negative controls. The immunocomplex samples were denatured in sample buffer plus DTT described as above before immunoblotting was performed against second primary antibodies,^35^ such as CD81 (mouse), with regular protein sample input as a positive control. The experiments were repeated at least 3 times. Phosphorylation and ubiquitination of the target proteins were also performed by 2‐step procedures, as indicated, including pulling down with specific antibodies of the target proteins and blotting with the antibodies against the modifications, or vice versa.

### 2.8 Preparation of membrane fractions with differential centrifugations

In order to observe the role of CD81 in the membranous distribution of NKCC2 and NCC, kidney samples were subjected to differential centrifugations to obtain membrane fractions as previously reported.^36^ At 4°C, the supernatants from the homogenates after centrifugation at 4000× *g* were subjected to two subsequent centrifugations: at 17 000× *g* for 20 min to obtain a high‐density fraction (17K pellets) and at 200 000× *g* for 60 min to obtain a low‐density fraction (200K pellets) with ultracentrifugation (OPTIMA XPN‐80, 70Ti rotor, Beckman, USA). The processes for protein concentration adjustment and sample preparation were the same as described in the immunoblotting and co‐immunoprecipitation sections above. The fractions were characterized by determining the enrichment of plasma membrane proteins (NKA and actin) and intracellular membrane vesicle proteins (GAPDH) in different fractions (supplemental results).

### 2.9 Extraction of urine exosomes for immunoblotting

Equal volume of urine samples was thawed and centrifuged, respectively, at 4000 *g* to remove cells and cellular debris and at 17 000 *g* to remove large membrane fractions, each for 10 min at 4°C. To increase exosome yields, the pellets were incubated at 37°C for 15 min in 2 mL of isolation buffer with DTT (400 mg) and centrifuged again for 10 min at 17 000 *g*. The combined supernatants were ultracentrifuged at 200 000 *g* for 1 h. The exosome‐enriched pellets were resuspended in 50 μL of the Isolation Solution mentioned above in immunoblotting plus 1 μg/mL leupeptin and 0.1 mg/mL PMSF and denatured in sample loading buffer with 1.5% DTT. The loading amounts for immunoblotting were adjusted based on equal urinary creatinine (C011‐2‐1, Nanjing Jiancheng Bioengineering Institute).^37^, ^38^, ^39^, ^40^

### 2.10 RNA extraction, reverse‐transcription and quantitative real‐time PCR (qPCR)

Total RNA was extracted from kidney cortex or cell suspension with RNA isolation kit (R401‐01, Vazyme) after ultra‐sonication. 1 μg of RNA was reverse‐transcribed into cDNA in a 20 μL of reaction (HiScript III RT SuperMix for qPCR, R323‐01, Vazyme). 1 μL of cDNA was applied in qPCR reaction (40 cycles) containing 20 μL of AceQ Universal SYBR qPCR Master Mix (Vazyme) and 10 nM of forward (F, 5′‐3′) and reverse (R, 3′‐5′) primers on a QuantStudio3 Applied Biosystems.^35^ Melting curves were performed to prove that the amplified double‐stranded DNAs were single discrete species. The primers were listed in Table S1 of the supplement file.

### 2.11 Measurements of serum and urine

The aldosterone in urine was measured with Aldosterone ELISA Kit (501090, Cayman). The concentrations of electrolytes, creatinine, and protein/albumin were measured using automatic biochemical analyzers (BS‐2000M, Mindray for serum, Cobas 6000 for urine).

### 2.12 Statistical analysis

Results are reported as mean ± SE (standard error of the mean). Significant differences between two groups were determined by the student *t*‐test. Significant differences among groups (>2) were determined by one‐way analysis of variance, followed by the Holm–Sidak test, as indicated. A value of *p* < .05 was considered significant while a value of *p* < .01 was considered very statistically significant.

## 3 RESULTS

### 3.1 Generation of rat model with PE

**FIGURE 1:**
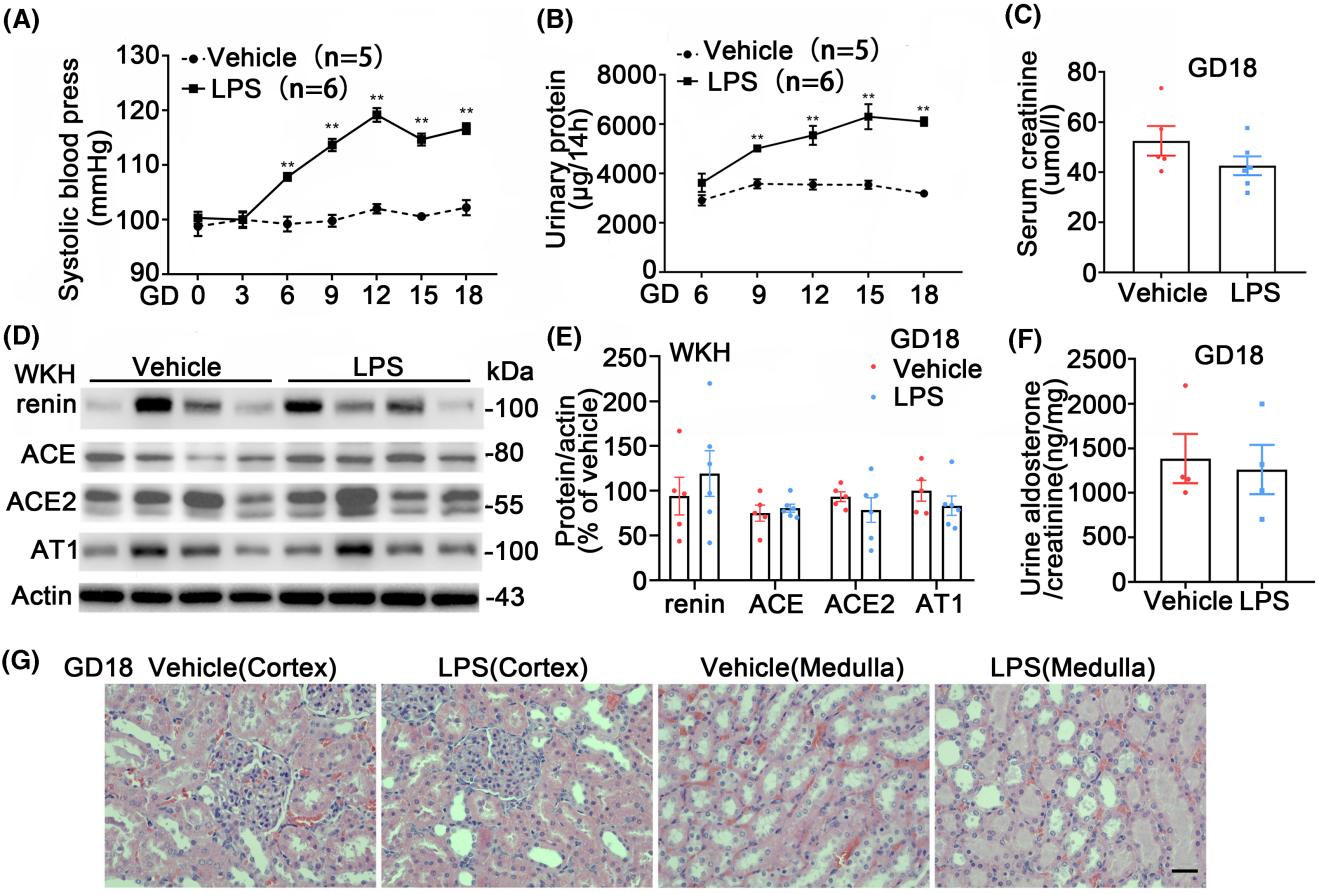
LPS‐induced preeclampsia (PE) model in rats and RAAS components on GD18. (A) Systolic blood pressure (SBP) of the PE rats. A single ultra‐low dose of lipopolysaccharide (LPS, 0.5 μg/kg) or vehicle (saline) was applied to female pregnant Sprague–Dawley rats (age: 10–12 weeks, BW: 268–280 g, restrained) on gestational day (GD) 5 by tail vein pump‐infusion. SBPs were monitored every 3 days (at 8–10 a.m., tail‐cuff, BP‐98A, Softron, Tokyo, Japan). LPS‐PE rats had higher SBPs than in vehicle rats since GD6 until GD18 when the rats were sacrificed. *n* = 5–6/group, student *t*‐test, **p < .01. Data were shown as mean ± SE, same as below. (B) Urinary protein. Urines were collected on GD18 in metabolic cages (8 p.m.–10 a.m.) for total protein measurement. The urinary protein excretions were higher in LPS than in vehicle rats since GD9 until GD18. *n* = 5–6/group, student *t*‐test, **p < .01. (C) Serum creatinine concentration on GD18. There was no difference in serum creatinine levels between groups. *n* = 5–6/group, student *t*‐test. (D) Immunoblots of renin, ACE, ACE2, AT1, and actin in whole kidney homogenates (WKH) on GD18. ACE, angiotensin‐converting enzyme; AT1, type 1 angiotensin II receptor. (E) Densitometry analyses of renin, ACE, ACE2, and AT1in WKH from LPS or vehicle rats. *n* = 5–6/group, student *t*‐test. (F) Urinary aldosterone excretion corrected by urine creatinine. It was not altered. *n* = 4/group, student *t*‐test. (G) The kidney sections of cortex and medulla stained with H&E. No obvious glomerulus cell proliferation and renal tubule collapses were seen in the LPS‐PE rat kidneys. The stainings in renal glomeruli and tubules were similar between groups. Magnification: 400, scale bar = 20 μm.

After LPS injection on GD5, higher systolic blood pressures (SBP) (Figure 1A) were seen in the LPS rats than in the vehicle rats since GD6 until GD18. The urinary protein excretions (Figure 1B) were higher in LPS rats than in vehicle rats since GD9 until GD18. The changes of SBP and proteinuria in LPS‐PE rats were similar as reported previously.^29^, ^31^, ^32^ CD81 protein abundances in original urines were similar between the two groups at all days while the abundances in both groups tended to rise with gestation days and were higher at GD18 relative to their baselines (Figure S1). On GD18 serum creatinine levels were similar between groups (Figure 1C). Body weight, urine output, serum potassium concentration, urine potassium excretion, and creatinine clearance were not altered; there were higher levels of urinary sodium/chloride excretion but lower serum sodium/chloride concentrations in LPS than in vehicle rats indicating that a negative salt balance was achieved, possibly due to an expansion of extracellular fluid volume toward the blunted pressure natriuresis^10^ in the model rats (Table S2). The fetus numbers (13.6 ± 0.31 vs. 15.4 ± 0.37, *p* < .05) and weights (2.12 ± 0.01 vs. 2.3 ± 0.02, g/fetus, *p* < .001) were lower in LPS‐PE than in vehicle rats (student *t*‐test). The components of intrarenal RAAS, including renin, angiotensin‐converting enzyme (ACE) and ACE2 in whole kidney homogenates (WKH), and urine aldosterone excretion, were also similar between groups (Figure 1D–F). The appearances of renal glomeruli and tubules, stained with H&E, in the kidney cortex and medulla were also similar between groups. No obvious glomerulus cell proliferations and renal tubule collapses were seen in the LPS‐PE rat kidneys (Figure 1G).

### 3.2 CD81, NKCC2, and NCC in kidneys on GD14 and CD81 in multiorgans on GD18

**FIGURE 2:**
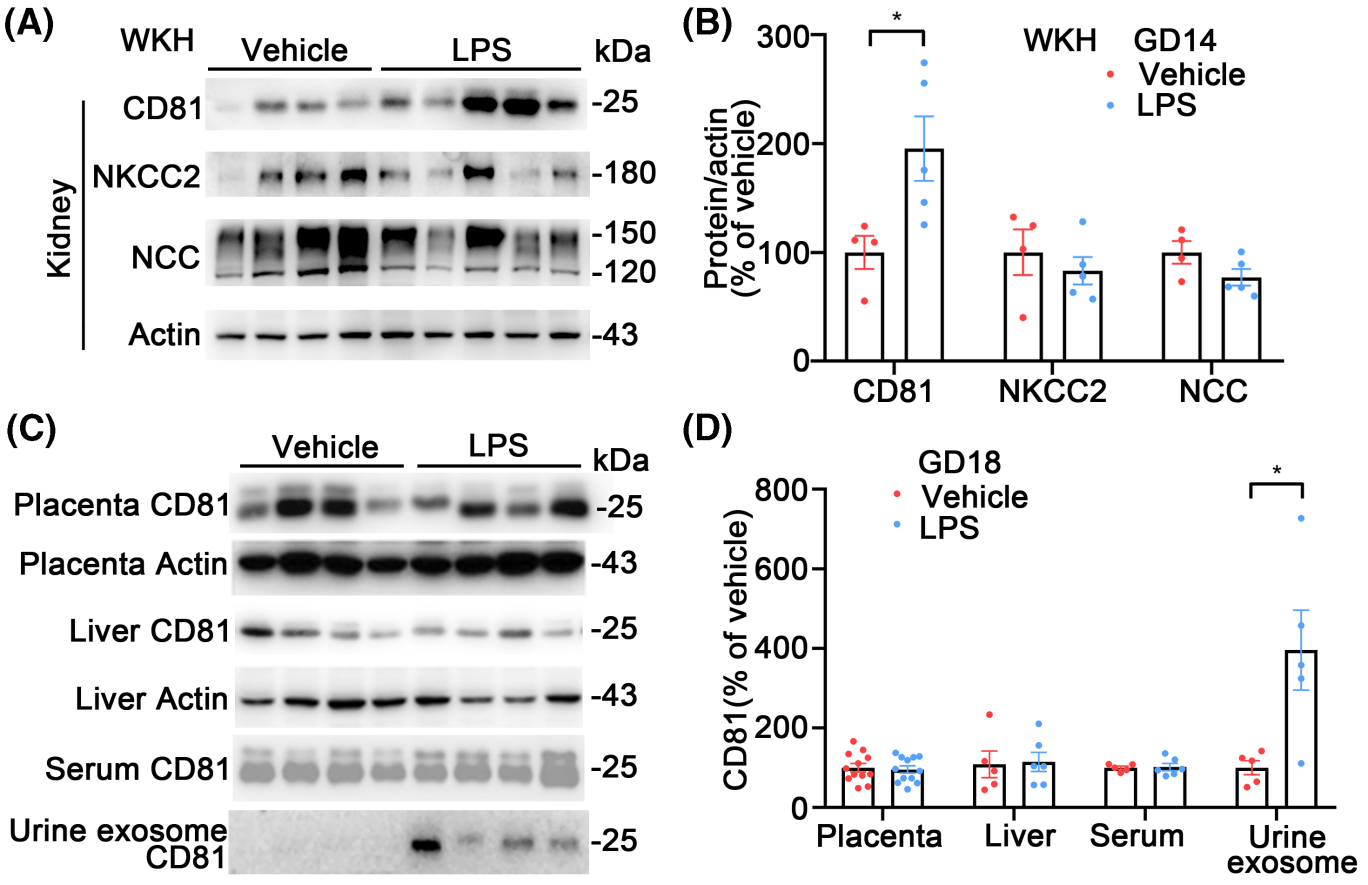
Renal CD81, NKCC2, and NCC on GD14, CD81 in placenta, liver, serum, and urine exosomes on GD18. (A) Immunoblots of renal CD81, NKCC2, NCC, and actin in WKH from LPS or vehicle rats on GD14. (B) Densitometry analyses of renal CD81, NKCC2, and NCC corrected by actin from LPS and vehicle rats on GD14. Renal CD81 protein abundance was higher in LPS rats than in vehicle rats while NKCC2 and NCC were not altered. *n* = 4–5/group, *p < .05, student *t*‐test. (C) Immunoblots of CD81 and actin in the homogenates of placentas and livers, and CD81 in sera and urine exosomes from LPS or vehicle rats on GD18. (D) Densitometry analyses of CD81 in the placentas, livers, sera, and urine exosomes from LPS or vehicle rats on GD18. Urine exosome CD 81 was higher in LPS rats than vehicle. Placentas: *n* = 3/rat, *n* = 12/group; Other organs: *n* = 5–6/group, *p < .05, student *t*‐test.

In order to explore whether CD81 was upregulated prior to the sodium transporters, additional rats were sacrificed on GD14. CD81 abundances in WKH were higher in LPS than in vehicle rats while NKCC2 and NCC were not altered in the LPS‐PE group relative to the vehicle (Figure 2A,B). The other sodium transport proteins, RAAS components, and proinflammation cytokines were not altered in the kidneys from the PE rats (Figure S2A,B). On the rats of GD18, CD81 in multiorgans was also studied. CD81 protein abundances in the placentas and livers from LPS‐PE rats were not altered relative to vehicle rats; serum CD81 levels were also similar between groups; however, CD81 abundances were higher in the urine exosomes from LPS‐PE rats compared with the vehicle group (Figure 2C,D), consistent with the alterations of CD81 in kidneys.

### 3.3 Protein abundance and mRNA expression of NKCC2 and NCC in LPS‐PE rats on GD18

**FIGURE 3:**
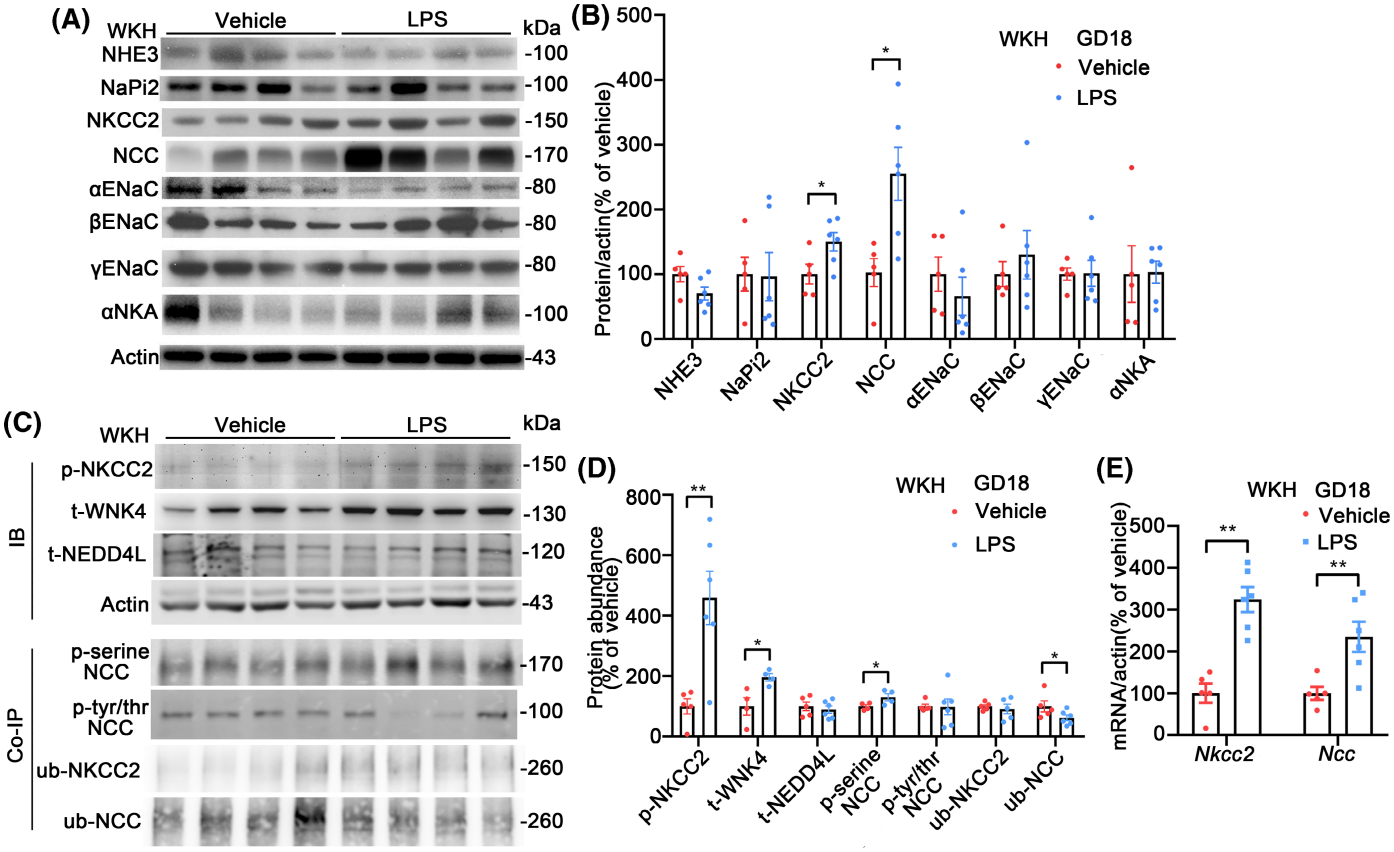
Profiled sodium transporters, channels, and pump along the nephron on GD18. (A) Immunoblots of renal sodium transporters, channels, pump, and actin. The samples were immunoblotted in whole kidney homogenates (WKH) from LPS or vehicle rats. (B) Densitometry analyses of sodium transporters, channels, and pump corrected by actin in WKH from LPS or vehicle rats. The protein abundances of NKCC2 and NCC were greater in LPS than in vehicle rats. *n* = 5–6/group, *p < .05, student *t*‐test. (C) Immunoblots of phosphorylated‐NKCC2 (p‐NKCC2), total WNK4, and NEDD4L (t‐WNK4 and t‐NEDD4L) and co‐immunoprecipitation (Co‐IP) blots of p‐serine NCC, phosphorylated‐tyrosine/threonine NCC (p‐tyr/thr NCC), ubiquitinated NKCC2, and NCC (ub‐NKCC2 and ub‐NCC), in WKH from LPS or vehicle rats. Ub‐NCC and p‐serine NCC were immunoblots against the modifications in the co‐immunoprecipitation complexes by NCC pull‐downs while Ub‐NKCC2 and p‐tyr/thr NCC were immunoblots of the sodium transporters in the co‐immunoprecipitation complexes by pull‐downs with the antibodies against the modifications. (D) Densitometry analyses of p‐NKCC2, t‐WNK4, t‐ NEDD4L, p‐serine NCC, p‐tyr/thr NCC, ub‐NKCC2, and ub‐NCC in WKH from LPS or vehicle rats. p‐NKCC2, t‐WNK4, and p‐serine NCC were higher, but ub‐NCC was lower in the LPS than in the vehicle group. ub‐NKCC2 and t‐NEDD4L were similar between the groups. *n* = 5–6/group, *p < .05, **p < .01, student *t*‐test. (E) mRNA relative levels of the renal sodium cotransporters in WKH by qPCR. The mRNA expressions of *Nkcc2* and *Ncc* were higher in the LPS than in the vehicle group. *n* = 5–6/group, **p < .01, student *t*‐test.

Among the major sodium transport proteins, the protein abundances of NKCC2 and NCC in WKH were greater in LPS (Figure 3A,B) than in vehicle rats. The renal phosphorylated NKCC2 (p‐NKCC2) and NCC (p‐serine NCC), by co‐immunoprecipitation (Co‐IP), were also higher together with WNK4 in the LPS group than in the vehicle group (Figure 3C,D). The phosphorylated threonine/tyrosine NCC, by Co‐IP, was similar between groups. The ubiquitination of NCC, but not NKCC2, by Co‐IP, was lower in the LPS group while NEDD4L, one of the neural precursor cell expressed developmentally down‐regulated proteins, was similar between the groups, indicating an involvement of NEDD4L independent ubiquitination mechanism (Figure 3C,D). Since there were no direct antibodies against ubiquitinated target proteins, 2‐step Co‐IP was used to determine the protein modifications with negative/positive controls (Figure S3A). There were higher relative mRNA levels of *Nkcc2* and *Ncc* found in the WKH of LPS rats than that of vehicle rats (Figure 3E). The mRNA expressions of other sodium transporters were not altered.

### 3.4 Renal CD81 protein abundance in LPS‐PE rats on GD18 and interaction of CD81 with NKCC2 and NCC in kidney

**FIGURE 4:**
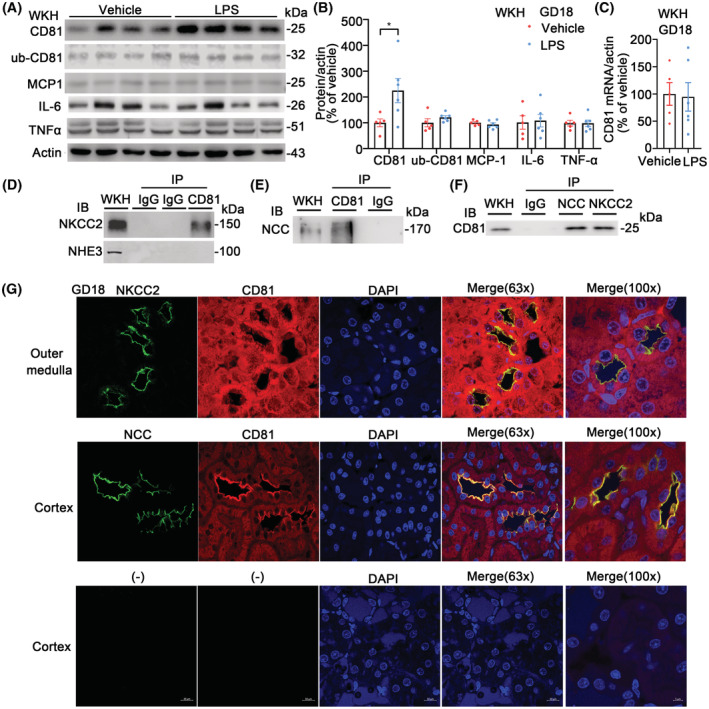
Total and ubiquitinated CD81 and proinflammation cytokines in LPS kidneys on GD18 and the interaction of CD81 with NKCC2 and NCC in rat kidneys. (A) Immunoblots of CD81, ub‐CD81, MCP1, IL‐6, TNF‐α, and actin from whole kidney homogenates of LPS or vehicle rats. ub‐CD81 was an immunoblot of ubiquitin in the co‐immunoprecipitation complexes from CD81 pull‐downs (Co‐IP). (B) Densitometry analysis of CD81, ub‐CD81, MCP1, IL‐6, and TNF‐α in WKH from LPS or vehicle rats on GD18. CD81 protein abundance was higher in LPS than in vehicle rats. *n* = 5–6/group. *p < .05, student *t*‐test. (C) Renal *Cd81* mRNA in WKH of LPS‐induced PE rats on GD18. There was no statistical difference between groups. Data were shown as mean ± SE, *n* = 5–6/group, student *t*‐test. (D) Co‐immunoprecipitation of CD81 with NKCC2 or NHE3 from normal rat kidney homogenates. NKCC2 (rabbit polyclonal antibody), not NHE3 (chicken polyclonal antibody), was detected in the co‐immunoprecipitation complexes pulled down with antibody against CD81 (mouse monoclonal) using mouse IgG as a negative control and WKH input as a positive control. (E) Co‐immunoprecipitation of CD81 with NCC from rat kidney homogenates. NCC (rabbit polyclonal antibody) was detected in the co‐immunoprecipitation complexes pulled down by antibody against CD81 (mouse monoclonal) with mouse IgG as a negative control and WKH input as a positive control. (F) Co‐immunoprecipitation of NKCC2 or NCC with CD81 from rat kidney homogenates. CD81 (mouse monoclonal antibody) was detected in the co‐immunoprecipitation complexes pulled down by antibody against NKCC2 (rabbit polyclonal antibody) or NCC (rabbit polyclonal antibody) with rabbit IgG as a negative control and WKH input as a positive control. (G) Confocal images of double staining for CD81 with NKCC2 or NCC in rat kidneys. Upper: The immunofluorescent staining of NKCC2 (green, rabbit, 1:200) was located in the apical membrane of renal tubules in the outer medulla indicating that the tubules were thick ascending limbs of the loop of Henle. The immunofluorescent staining of CD81 (red, mouse, 1:200) was diffused but colocalized with NKCC2 at the luminal membrane (yellow areas in 63× merge image and 100× merge image). Nuclei were stained by DAPI (blue). Middle: The immunofluorescent staining of NCC (green, rabbit, 1:200) was located in the apical membrane of renal tubules in the cortex indicating that the tubules were distal convoluted tubules. The immunofluorescent staining of CD81 (red, mouse, 1:200) was concentrated and colocalized with NCC at the luminal membrane (yellow areas in 63× merge image and 100× merge image). Nuclei were stained by DAPI (blue). Bottom: There was no staining in the kidney section treated with secondary antibodies against rabbit and mouse IgG (negative controls). Magnification: 630 or 1000, scale bar = 10 μm in 63× image; scale = 5 μm in 100× image.

In WKH the protein abundance of CD81 was higher in LPS rats while monocyte chemoattractant protein‐1 (MCP1), interleukin 6 (IL‐6), and tumor necrosis factor‐α (TNF‐α) were similar between LPS and vehicle rats. The ubiquitinated CD81 was also not altered (Figure 4A,B). There were no changes in *Cd81* mRNA (Figure 4C). The melting curves of *Cd81*, *Nkcc2*, *Ncc*, and β‐actin indicated that the amplified double‐stranded DNAs were single discrete species (Figure S4). The specificity of CD81 antibody was characterized by the immunoblotting against a recombined human CD81 peptide (Figure S5).

NKCC2 and NCC, but not NHE3 (Figure 4D,E), were detected in the co‐immunoprecipitated complexes with CD81 pull‐downs from whole kidney homogenates, while CD81 was also found in the co‐immunoprecipitated complexes with NKCC2 or NCC pull‐downs (Figure 4F). NKCC2 and NCC were not detected in the pull‐downs by CD63, another transmembrane 4 superfamily protein (Figure S6). Those results indicate that the interactions of renal CD81 with NKCC2 and NCC may be protein‐specific. In the outer medulla of rat kidney (Figure 4G, upper) the immunofluorescent staining of NKCC2 (green) was located in the apical membrane of renal tubules indicating that the tubules were thick ascending limbs of the loop of Henle (TAL). The immunofluorescent staining of CD81 (red) was diffused but colocalized with NKCC2 in the apical membrane (yellow areas in 63× merge image and 100x merge image). In the cortex of rat kidney (Figure 4G, middle), the immunofluorescent staining of NCC (green) was located in the apical membrane of renal tubules indicating that the tubules were distal convoluted tubules (DCT). The immunofluorescent staining of CD81 (red) was concentrated and colocalized with NCC at the luminal membrane (yellow areas in 63× merge image and 100× merge image). The co‐immunoprecipitation and colocalization of CD81 with NKCC2 and NCC suggested that CD81 was interacted with the two major apical sodium cotransporters located in TALs and DCTs, respectively.

### 3.5 Association of CD81 with NKCC2 and NCC in differential membrane fractions from LPS‐ PE rats on GD18

**FIGURE 5:**
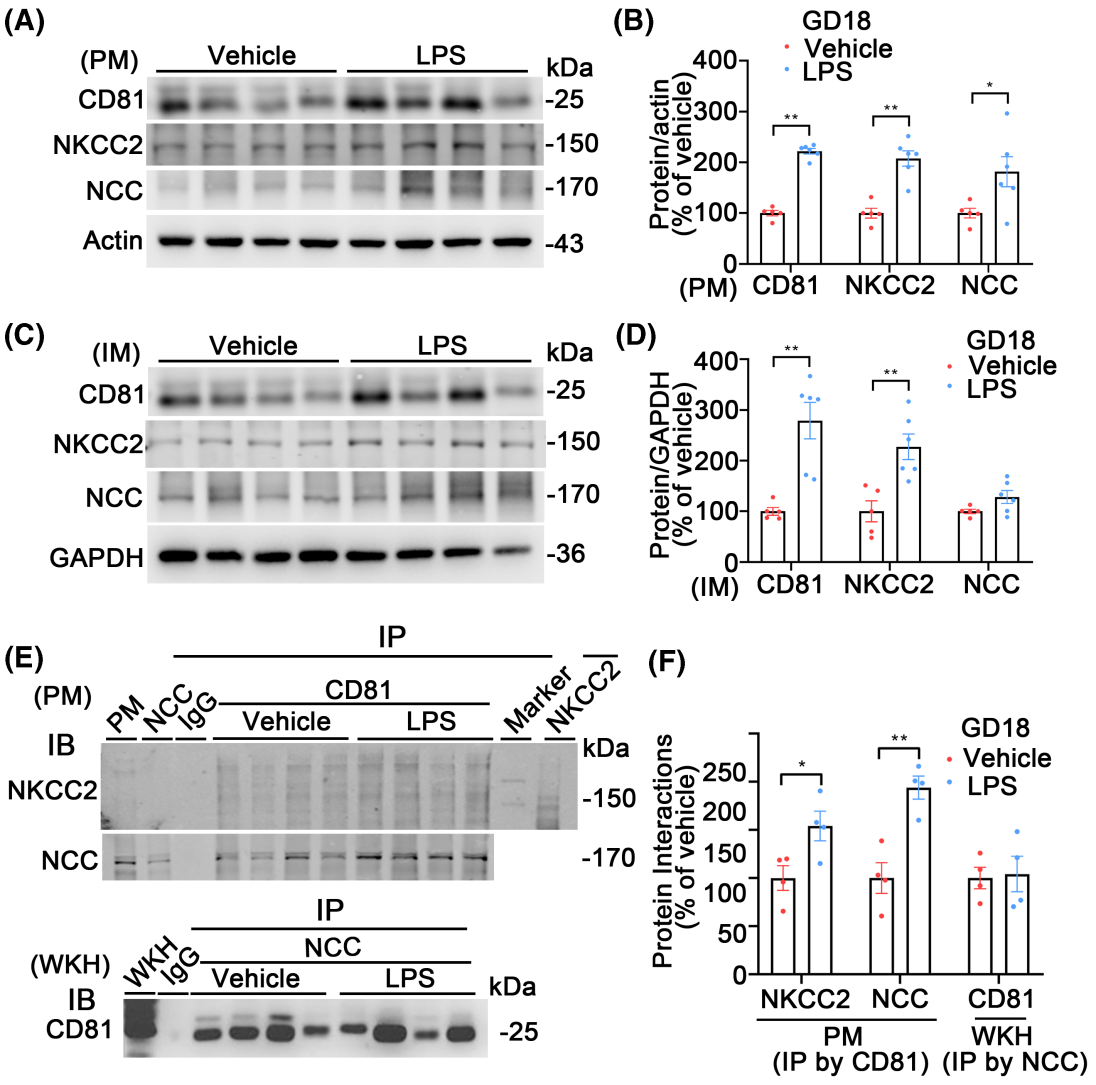
Association of renal CD81 with NKCC2 and NCC in differential membrane fractions in LPS rat model on GD18. (A) Immunoblots of renal CD81, NKCC2, NCC, and actin in the plasma membrane (PM) at 17 000 *g* pallets from LPS or vehicle rats. (B) Densitometry analyses of renal CD81, NKCC2, and NCC in PM from LPS or vehicle rats. Renal CD81, NKCC2, and NCC were higher in PM from the LPS group than that from vehicle. *n* = 5–6/group, *p < .05, **p < .01, student *t*‐test. (C) Immunoblots of renal CD81, NKCC2, NCC, and GAPDH in intracellular (vesicle) membrane (IM) at 200 000 *g* pellets from LPS or vehicle rats. (D) Densitometry analyses of renal CD81, NKCC2, and NCC in IM from LPS or vehicle rats. CD81 and NKCC2 were higher in IM from the LPS group than the vehicle group. NCC was slightly higher but had no significant difference at IM between the groups. *n* = 5–6/group, **p < .01, student *t*‐test. (E) Co‐immunoprecipitation of renal CD81 with NKCC2 and NCC in PM from vehicle and LPS rats. Upper 2 blots: the PM‐enriched fractions were pulled down with CD81 mouse monoclonal antibody; the complexes were immunoblotted with rabbit polyclonal antibodies against NKCC2 or NCC, respectively; nonspecific mouse IgG was used as a negative control while PM input and corresponding antibody‐pull downs as positive controls. Bottom: CD81 (mouse monoclonal antibody) was the immunoblot of the co‐immunoprecipitation complexes from NCC pull‐downs in whole kidney homogenates (WKH) with rabbit IgG as a negative control and kidney input as a positive control. (F) Densitometry analyses of the interactions of CD81 with NKCC2 and NCC in vehicle and LPS rats. In CD81 pull‐downs from PM‐enriched fractions, the co‐immunoprecipitations of CD81 with NKCC2 or NCC were higher in the LPS group than in the vehicle group while the co‐immunoprecipitations of CD81 with NCC were similar between groups in WKH. *n* = 4/group, *p < .05, **p < .01, student *t*‐test.

Since CD81 is a member of transmembrane 4 superfamily proteins, the associations of CD81 with NKCC2 and NCC were studied in the differential membrane fractions prepared from rat kidneys. The LPS‐PE rats had a higher abundance of NCC in 17K plasma membrane‐enriched fractions (PM) but not in 200K intracellular vesicle‐enriched membrane fractions (IM) while CD81 and NKCC2 were higher in both fractions than vehicle groups (Figure 5A–D). More importantly, in PM, CD81 co‐immunoprecipitations with NKCC2 and NCC were higher in LPS than in vehicle while co‐immunoprecipitations of NCC with CD81 were not altered in WKH (Figure 5E,F). Those findings indicate that the higher CD81 may mediate the redistribution of NKCC2 and NCC into the plasma membranes in LPS‐PE rats, thus enhance their functions on sodium transport. CD81 was enriched together with NKCC2 and NCC in PM of the 17K pellets. The 17K PM fractions were enriched with CD81, NKCC2, and NCC and characterized by higher plasma membrane proteins actin and NKA and lower intracellular vesicle protein GAPDH relative to WKH (Figure S7A,B).

### 3.6 NCC and CD81 in mDCT cells treated with LPS and Cd81‐siRNA

**FIGURE 6:**
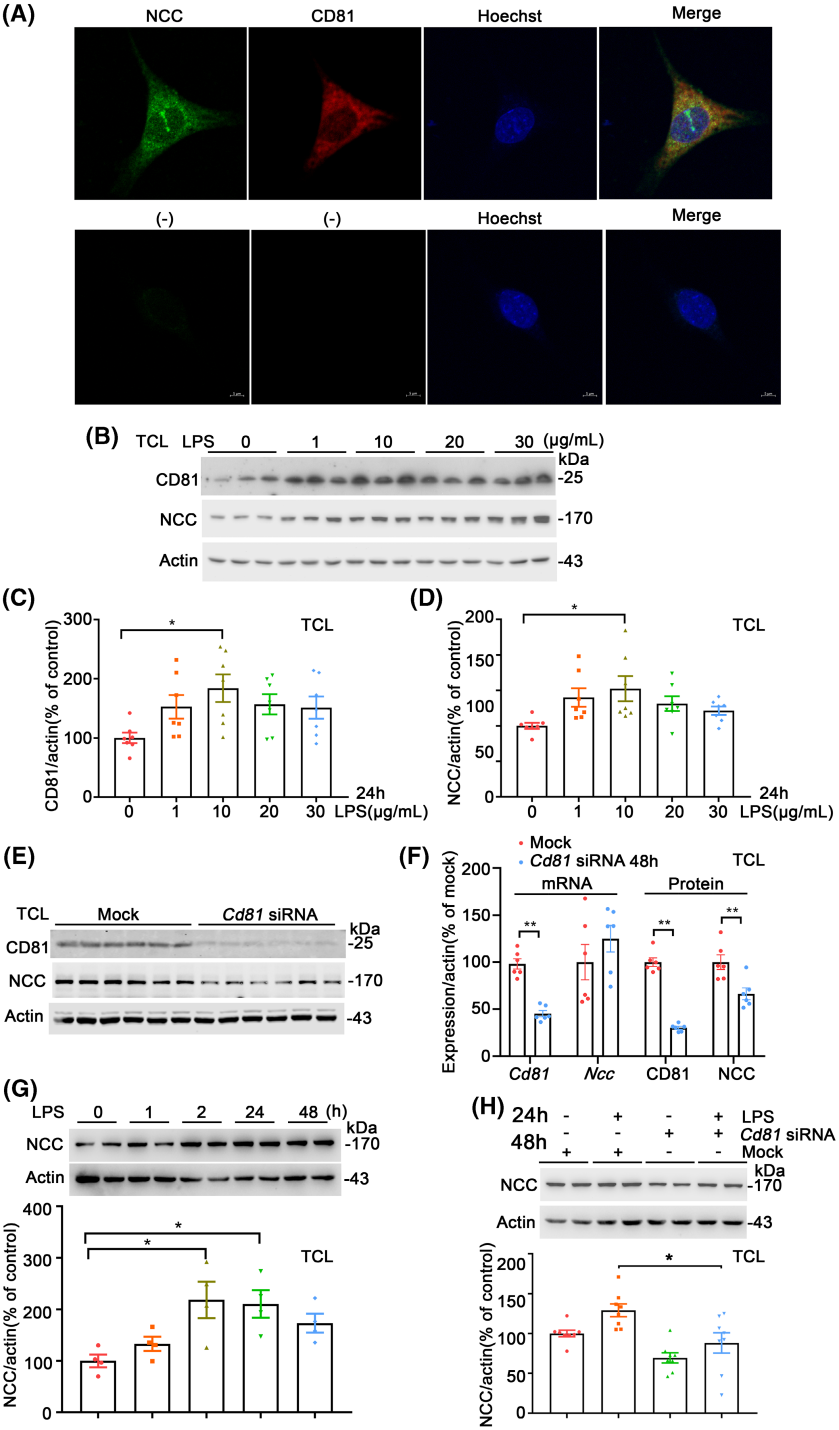
NCC and CD81 in cultured mDCT cells treated with LPS or/and C*d81*‐siRNA. (A) Immunofluorescence of NCC and CD81 with confocal microscopy in cultured mDCT cells. The immunofluorescent staining of NCC (green, rabbit, 1:200) was located in the sub‐cellular membrane and cytoplasm while CD81 (red, mouse, 1:200) was colocalized with NCC shown as yellow spots in the merged image. Nuclei were stained with Hoechst in blue. Magnification: 1000, scale bar = 5 μm. mDCT: mouse distal convoluted tubule. (B) Immunoblots of CD81, NCC, and actin in mDCT cells treated by LPS at different concentrations for 24 h. Total cell lysates (TCL) from mDCT cells were used, same as below. (C,D) Densitometry analyses of CD81 and NCC in mDCT cells treated with LPS at different concentrations for 24 h. At 10 μg/ml, LPS‐induced higher protein abundances of CD81 and NCC than vehicle. *n* = 6/group, *p < .05, one‐way ANOVA, Holm–Sidak test. The data were shown as mean ± SE, same as below. (E) immunoblots of CD81, NCC, and actin in mDCT cells treated with *Cd81*‐siRNA or mock‐siRNA (50 nM) for 48 h. (F) mRNA expressions of *Cd81* and *Ncc* measured by qPCR and protein expressions of CD81 and NCC by immunoblotting in mDCT cells treated with *Cd81*‐siRNA for 48 h. The inhibition of *Cd81*, *not Ncc*, was confirmed at mRNA levels. Both CD81 and NCC were lower in the siRNA‐treated cells than mock at protein levels. *n* = 6/group, **p < .01 versus the mock, student *t*‐test. (G) Immunoblots and densitometry analyses of NCC in mDCT cells treated with LPS in 0, 1, 2, 24 and 48 h. mDCT cells were treated with LPS for 10 μg/mL. NCC protein abundances were higher in LPS‐stimulated cells on 2 and 24 h than baseline. *n* = 4/group, *p < .05, one‐way ANOVA, Holm–Sidak test. (H) Immunoblots and densitometry analyses of NCC in mDCT cells treated with *Cd81*‐siRNA and LPS. mDCT cells were treated with *Cd81*‐siRNA or mock (50 nM) for 24 h, then LPS (10 μg/mL) or vehicle was added into the co‐incubations for another 24 h. *Cd8*1‐siRNA inhibited NCC protein and prevented the LPS‐induced elevation of NCC. *n* = 8/group, *p < .05, one‐way ANOVA, Holm–Sidak test.

All mouse renal distal convoluted tubule (mDCT) cells under confocal microscopy exhibited a positive immunostaining of NCC, the sodium transporter only expressed in DCT (Figure S8A). NCC immunofluorescent staining (green) was located in the sub‐cellular membrane and cytoplasm while CD81 (red) was colocalized with NCC shown as yellow spots in the merge image (Figure 6A). The protein abundances of CD81 and NCC were greater in the cell group treated with LPS at a concentration of 10 μg/mL for 24 h (Figure 6B–D). LPS at 10 μg/mL had no effects on the toxicity and viability of the cells measured with LDH and CCK‐8 kits (Figure S8B,C).

In order to determine the direct effect of CD81 on NCC in the mDCT cells with both proteins expressed naturally, *Cd81*‐siRNA or mock were transfected to the cells for 48 hrs. The protein abundance of CD81 and mRNA expression of *Cd81* were lower remarkably with siRNA treatment (Figure 6E,F). More interestingly, NCC protein abundance was also lower in *Cd81*‐siRNA group while mRNA expression of *Ncc* was not altered (Figure 6E,F). Higher NCC was induced by LPS at 10 μg/mL for 2 hr and 24 h relative to baseline (Figure 6G). The LPS‐induced elevation of NCC was prevented by the *Cd81*‐siRNA pretreatment (Figure 6H). Those findings further indicated a direct inhibition of *Cd81‐*siRNA on NCC protein abundance and confirmed a positive regulation of CD81on NCC in vitro in the presence or absence of LPS stimulation.

### 3.7 CD81 in original urines and CD81, NKCC2, and NCC in urine exosomes from the patients with PE

**FIGURE 7:**
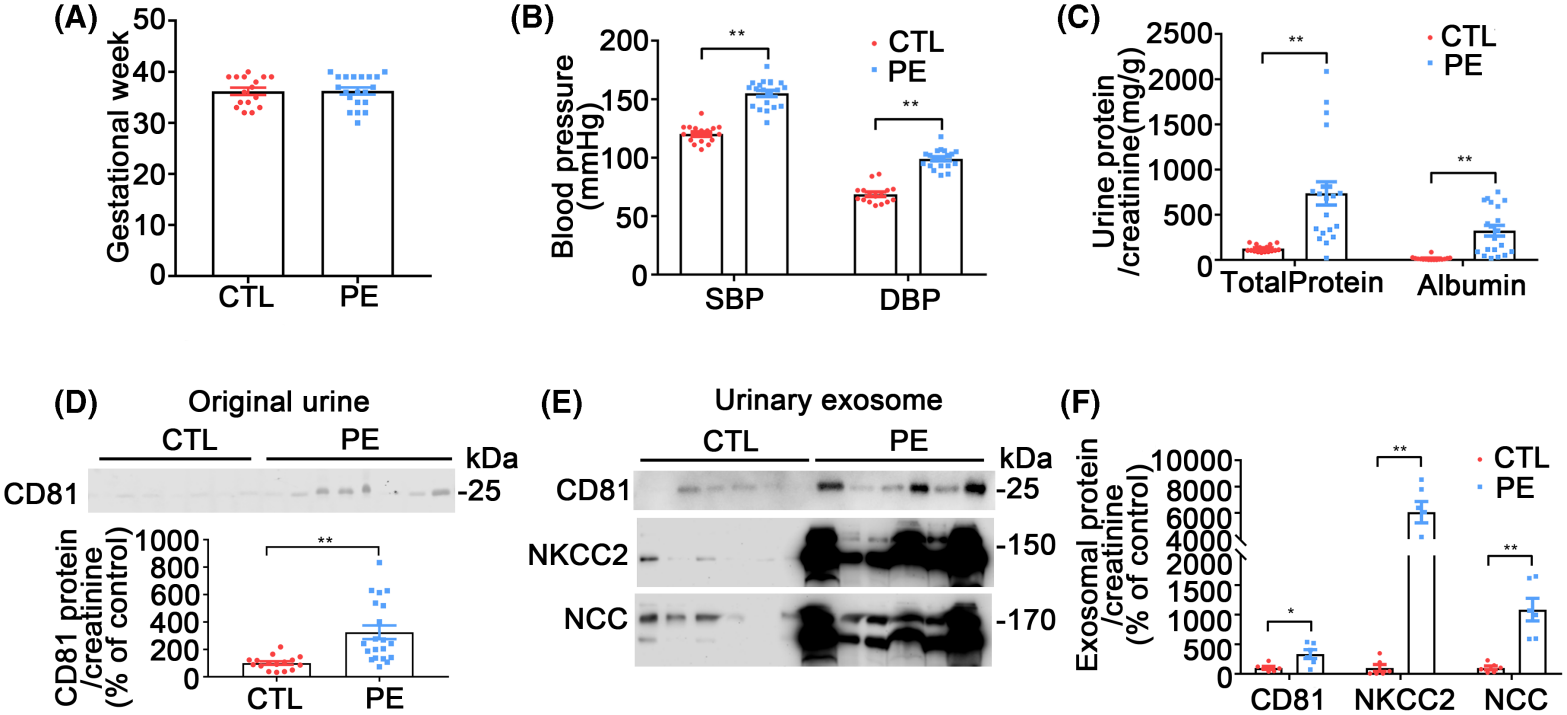
The studies on the patients with PE. (A) Gestational weeks of the human subjects including untreated in patients with PE and healthy pregnant women as controls (CTL). There was no difference between the groups. Data were shown as mean ± SE, same as below, *n* = 16–20/group. (B) Blood pressures. SBP and DBP were higher in the PE group than in the CTL. *n* = 16‐20/group, **p < .01, student *t*‐test. (C) Urinary excretion of total protein and albumin. Urine total protein and albumin, corrected by urine creatinine, were higher in the PE relative to the CTL group. *n* = 16–20/group, **p < .01, student *t*‐test. (D) Immunoblots and densitometry analyses of CD81 in original urine samples. CD81 protein was higher in the PE relative to CTL group. The volumes of loading were corrected by urine creatinine, *n* = 16–20/group, **p < .01, student *t*‐test. (E) Immunoblots of CD81, NKCC2, and NCC in urine exosomes. The volumes of loading were corrected by urine creatinine measurements. (F) Densitometry analyses of CD81, NKCC2, and NCC in urine exosomes. Urine exosomal CD81 was higher in the PE group than in controls. The target bands of NKCC2 and NCC were both strong in the PE group while they were weak in the CTL group. *n* = 6/group, *p < .05, **p < .01, student *t*‐test.

The human subjects included in this study had similar gestational weeks between the CTL (*n* = 16) and PE (*n* = 20) groups (Figure 7A). SBP and DBP were higher in PE pregnant women than in normal ones (CTL) (Figure 7B). Urinary excretions of protein and albumin corrected by creatinine were higher in the PE group than CTL group (Figure 7C,D). The body weight, height, heart rate, respiration rate, gravidity, and parity were similar in the 2 groups except for the controls were slightly younger than PE (Table S3). There were no differences in serum protein, albumin, urine/serum electrolytes, and creatinine between PE and normal pregnant women (Table S4). In order to simplify its quantification for clinical application in the future, CD81, NKCC2, and NCC were measured in the original urine samples without ultracentrifugation. CD81 protein was higher in the PE relative to the CTL group (Figure 7D), for NCC, 1 out of 16 (6.25%) in controls was positive while 10 out of 20 in PE were positive (50%), for NKCC2, all controls were negative while 8 out of 20 in PE were positive (40%) (Figure S9). In the urine exosome extracts, CD81, NKCC2 (60 times more), and NCC (10 times more) were higher in the PE group than in the CTL group (Figure 7E,F).

## 4 DISCUSSION

Our findings explore a novel mechanism in the development of PE. In addition to hypertension and proteinuria developed with the pregnancy, CD81 protein abundances in both groups tended to rise and were higher at GD18 relative to GD0. Higher protein–protein interactions of renal CD81 with NCC or NKCC2 were identified by co‐immunoprecipitation and colocalization in LPS‐PE rats than controls. There was an upregulation of renal CD81 on GD14, which was 4 days prior to the alterations in protein abundances and phosphorylations of NKCC2 and NCC in LPS‐PE rats. The potential protein–protein interactions of CD81 with NCC and NKCC2 were confirmed in kidneys through co‐immunoprecipitation and colocalization studies; the higher protein–protein interactions of CD81 with NCC and NKCC2 were found in the plasma membrane‐enriched fractions prepared from the LPS‐induced PE rat kidneys. The interaction of CD81 with NCC was further confirmed in the immortalized mouse renal distal convoluted tubule cells (mDCT): silencing CD81 with siRNA lowered NCC and prevented the increase in NCC induced by LPS consequently, indicating that CD81 may positively regulate NCC. Those findings suggest that through protein–protein interaction an enhanced renal CD81 plays an important role in the upregulation of renal sodium transporters. The higher renal sodium transporters associated with CD81 may act to increase the sodium/water reabsorptions from the affected nephron segments and blunt pressure natriuresis, thus contributing to the pathogenesis of hypertension in PE. When we tested CD81 in urinary exosomes from PE patients, we observed higher CD81, NKCC2, and NCC relative to pregnant women not presenting with PE. Higher CD81 is also detected in the original urines from PE than controls. The detection of higher CD81 in urinary exosomes and original urines from PE patients is a novel finding. With further studies, measurements of urinary CD81, NKCC2, and NCC may be developed into a noninvasive diagnostic tool for PE. Since the 2 distal sodium cotransporters that CD81 interacts with are target proteins for 2 commonly used diuretics in antihypertensive treatment (bumetanide for NKCC2 and thiazide for NCC, respectively), the findings may facilitate the choice of a proper diuretics for the treatment of PE patients.

Prior research shows that several sodium transporters are involved in aldosterone escape, including NCC^8^ and NKCC2.^11^ The abundance of NKCC2 and NCC is known to be regulated by RAAS,^41^, ^42^ but we have not observed any alterations in RAAS components, including renal renin, ACE, ACE2, AT1, and urinary aldosterone excretion, in the LPS‐PE rats. An enhanced protein–protein interaction is found between renal CD81 with NKCC2 or NCC in the plasma membranes of LPS‐PE rats in vivo. Further interaction is evident in cultured mDCT cells, in which CD81 and NCC are expressed and colocalized naturally. The renal CD81 is also higher in LPS‐PE rats than controls on GD14, 4 days prior to the alterations of NKCC2 and NCC, suggesting that the changes of sodium transporters may be secondary to that of CD81. Thus, the higher abundance of NKCC2 and NCC may be RAAS‐independent but CD81‐associated in the LPS‐induced PE model. Although the upregulation of renal CD81 on NKCC2 and NCC may be important in the development of hypertension at late pregnancy, the current study cannot exclude the effects of original abnormalities in placentas and fetuses on kidney injuries.

CD81 is considered an exosome marker.^43^, ^44^ In the current PE model, we observed a higher CD81 in urine exosomes together with that in kidneys at GD18 although CD81 in original urine samples tended to rise with pregnancy but was not different between groups. In PE patients CD81 has been identified as one of the PE‐unique proteins in the syncytiotrophoblast microvesicles from the placentas^27^ and higher in the serum exosomes.^26^ However, we did not find higher CD81 abundances in placentas, livers, or sera in the LPS‐PE rats on GD18, indicating that the alterations of CD81 may be kidney‐specific. There was a heterogeneous distribution of the CD81 in differential rat kidney membrane fractions with the abundance highest in the intracellular vesicle (200K pellets) but negative in the intracellular soluble fractions (200K supernatants), suggesting that CD81 could be an intracellular membrane marker as well. CD81 was also enriched and expressed at higher abundances together with NKCC2 and NCC in the plasma membrane‐enriched fractions (17K pellets). It is possible that CD81 may keep the sodium transporters on the apical surface of the epithelial cells and promote its release together with sodium transporters into exosomes. Recent reports show that tetraspanins, including CD81, are involved in the migrations of the intraluminal vesicles from endosomes to exosomes across plasma membranes in the bilayers.^45^, ^46^ In LPS‐PE rat kidneys there was a simultaneous increase in CD81 with NKCC2 and NCC in the 17 K plasma membrane‐enriched fractions. In the same fraction, the higher protein–protein interactions of CD81 with NKCC2 and NCC were found by co‐immunoprecipitation. Thus, CD81 may play an important role in the recruitment of those transporters into their membrane domains and facilitate their functions. CD81 may also exert its regulation on the sodium transporters through exosomes, which will be studied in future. In addition, we found higher phosphorylations of NKCC2 and NCC together with WNK4, a specific activating enzyme for NKCC2 and NCC.^47^ But it remains to be determined in the future whether CD81 directly affects WNK4 or phosphorylation of the transporters. Taken together, the recruit of NKCC2/NCC in renal plasma membranes due to upregulations of CD81 and phosphorylations of NKCC2/NCC may blunt the inhibitions of those distal tubule sodium transporters triggered by the elevation of BPs (pressure natriuresis) and contribute to the sustained hypertension observed in the LPS‐induced PE rats.

We have reported that a single administration of 0.5 μg/kg LPS to pregnant rats on GD5, same as the current model, BP starting higher on GD6 and causes proteinuria starting on GD9; MCP1 and IL‐6 are elevated in the sera and placentas.^29^, ^48^ The role of CD81 in this model is not reported. In the current study, renal MCP1, IL‐6, and TNF‐α were not altered on GD18 (Figure 3A,B), consistent with the previous report.^48^ Therefore, CD81‐mediated inflammation response^48^ may not be responsible for the upregulation of renal sodium transport proteins in this model.

In LPS‐PE rats, we observed higher renal mRNA levels of *Nkcc2* and *Ncc*. Because *Ncc* mRNA is not altered by CD81 deletion in vitro, there may be CD81‐independent factors involved in the regulation.^49^ In addition, ubiquitination of NCC, not NKCC2, was lower in the PE rat model, suggesting that the higher abundance of NCC may be related to the reduction in ubiquitin‐induced protein degradation. Because NEDD4L, an E3 ubiquitin ligase for NCC ubiquitination,^50^, ^51^ was not altered, we will determine whether CD81 could directly affect the ubiquitination and degradation of NCC in future. Poly‐ubiquitination and clathrin‐mediated endocytosis of cell surface CD81 have been reported recently.^52^ The mechanism for the higher abundance of renal CD81 also remains to be explored since there are no changes in either CD81 ubiquitinations or *Cd81* mRNA levels with the rat‐PE model. The relevance of the CD81/NCC/NKCC2 interactions will be assessed in a mouse PE model that CD81 is over‐expressed in placenta.

In conclusion, CD81 interacts with NKCC2 and NCC in LPS‐PE rat kidneys and positively regulates NCC in cultured mouse renal distal convoluted tubule cells. The enhanced recruit of NKCC2 and NCC into renal plasma membranes in LPS‐PE rats links to upregulations of CD81. Those changes may contribute to the sustained hypertension observed in LPS‐PE model. Higher CD81 with NKCC2 and NCC in PE patients than controls are seen in original urines and urine exosomes and may be used as noninvasive biomarkers for PE.

## AUTHOR CONTRIBUTIONS

Ping Wang completed most experiments on kidney membrane fractions and the cells, figured out the data, and wrote the corresponding portions. Gangyi Zhu completed the most immunoblotting and immunostaining experiments on the GD18 rat model. Qiaozhen Wu completed clinical data collection and analyses, figured the data, and wrote the corresponding portions. Li Shen, Dan Liu, and Zhiyin Wang worked on the rat model for LPS infusions, BP measurements, and sample collections/analyses. Weiwan Wang completed the most experiments of co‐immunoprecipitation. Zhiyun Ren, Yutao Jia, and Mingda Liu performed the experiments and analyses for rat urine exosome, second set of the rat model and confocal images, respectively. Ying Xue performed partial immunoblots and analyses on CD81, NKCC2 and NCC in original urine samples from patients. Yali Hu and Daxi Ji participated in the designs of the studies on animal models and clinical patients and revised portions of the manuscript. Yanting Yu conceptualized the manuscript and did confocal microscopy, and wrote portions of the manuscript. Xiaoyan Wang conceptualized, designed the entire study, wrote/revised the manuscript critically, and is responsible for the integrity of all data included.

## FUNDING INFORMATION

This work is supported by grants from the National Natural Science Foundation of China (81900650 and 81970605), Natural Science Foundation of Jiangsu Province (BK20190128), and Nanjing Municipal Health Bureau (YKK20217). L. Shen, D. Liu, Z. Wang, and Y. Hu are supported by grants from the National Key R&D Program of China (2018YFC1004404), and National Natural Science Foundation of China (81771526).

## DISCLOSURES

All the authors declared no interest to disclose.

## Supporting information


Supporting information S1

## Data Availability

Source data are available for Figures 1, 2, 3, 4, 5, 6, 7. On reasonable request, data, analytic methods, and study materials will be made available to other researchers for the purpose of reproducing the results.
