# Supplementary material for: Renal CD81 interacts with sodium potassium 2 chloride cotransporter and sodium chloride cotransporter in rats with lipopolysaccharide‐induced preeclampsia
Source: FASEB J. 2023 Mar 24;37(4):e22834. doi: 10.1096/fj.202201546RR (PMC11977528; doi:10.1096/fj.202201546RR)
Supplement: Supplementary file 1 — Supporting information S1 [file FSB2-37-e22834-s001.pdf]

SUPPLEMENTAL FILE

RENAL CD81 INTERACTS WITH NKCC2 AND NCC IN RATS WITH LIPOPOLYSACCHARIDE-INDUCED PREECLAMPSIA

Ping Wang[1,2*], Gangyi Zhu[1*], Qiaozhen Wu[*3], Li Shen[4], Dan Liu[4], Zhiyin Wang[4], Weiwan Wang[1], Zhiyun Ren[1], Yutao Jia[2], Mingda Liu[1], Ying Xue[1], Daxi Ji[2], Yali Hu[4], Yanting Yu[1,2], and Xiaoyan Wang[1,2]

[1]The Core Laboratory for Clinical Research, BenQ Medical Center, The Affiliated BenQ Hospital of Nanjing Medical University, Nanjing, China.

[2]Department of Nephrology, BenQ Medical Center, The Affiliated BenQ Hospital of Nanjing Medical University, Nanjing, China.

[3]Department of Obstetrics and Gynecology, BenQ Medical Center, The Affiliated BenQ Hospital of Nanjing Medical University, Nanjing, China.

[4]Department of Obstetrics and Gynecology, Nanjing Drum Tower Hospital, Nanjing University Medical School, Nanjing, China.

* The authors have equal contribution to the manuscript.

<Xiaoyan Wang and Yanting Yu, The Core Laboratory for Clinical Research and Department of Nephrology, BenQ Medical Center, The Affiliated BenQ Hospital of Nanjing Medical University, Nanjing, China. Email: xiaoyan.wang@benqmedicalcenter.com and yuyanting@njmu.edu.cn>

S. Results

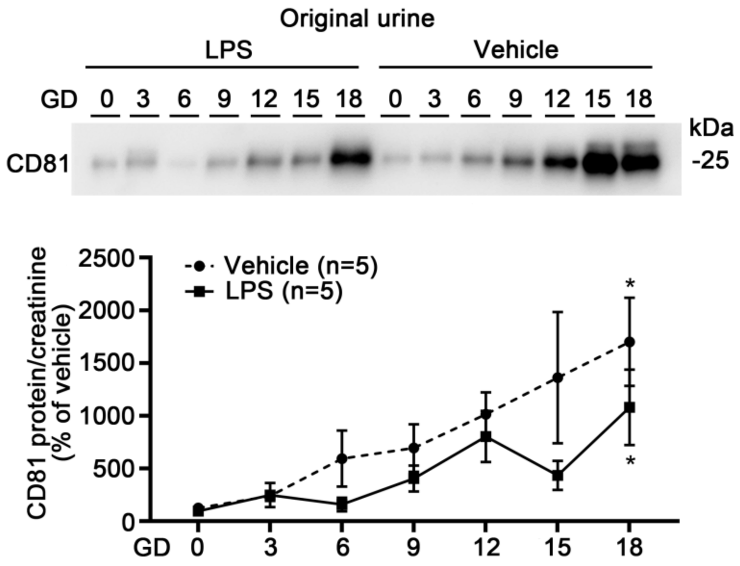

Figure S1. CD81 immunoblotting and densitometry analyses on the daily original urine samples from LPS and vehicle pregnant rats. There were no significant differences in CD81 protein abundances between the two groups at all days while the abundances in both groups tended to rise with gestation days and were higher at GD18 relative to their baseline. One-way Anova, Holm-Sidak test, n=5, *p<0.05 vs GD0.

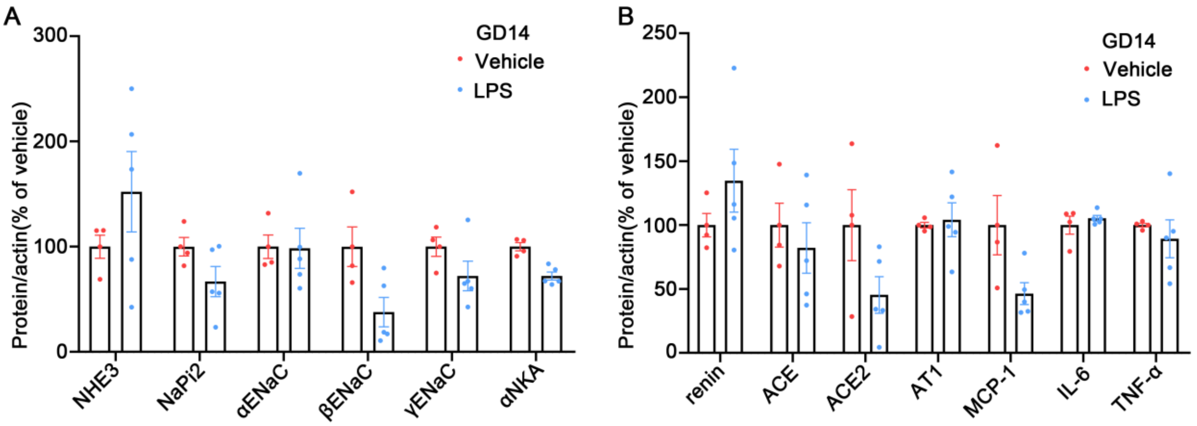

Figure S2. Densitometry analyses of sodium transporters, channels, and pump (A), RAAS and Inflammatory factors (B) corrected by actin in WKH from LPS or vehicle rats on GD14. The protein abundances were similar between groups. n=4-5/group, student t-test.

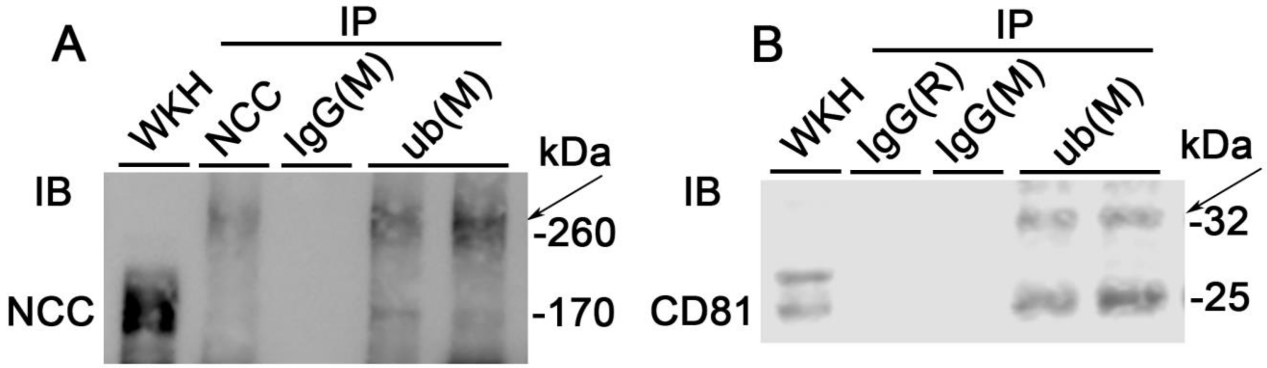

Figure S3. Co-immunoprecipitation of ubiquitin (ub) with NCC (A) and CD81 (B) from rat whole kidney homogenates (WKH). NCC (rabbit polyclonal antibody) and CD81 (mouse monoclonal antibody) were detected by immunoblotting (IB) in the co-immunoprecipitation (IP) complexes pulled-down with antibody against ubiquitin using mouse IgG as a negative control and WKH input as a positive control. The arrows point to the ubiquitinated-NCC and ubiquitinated-CD81 bands.

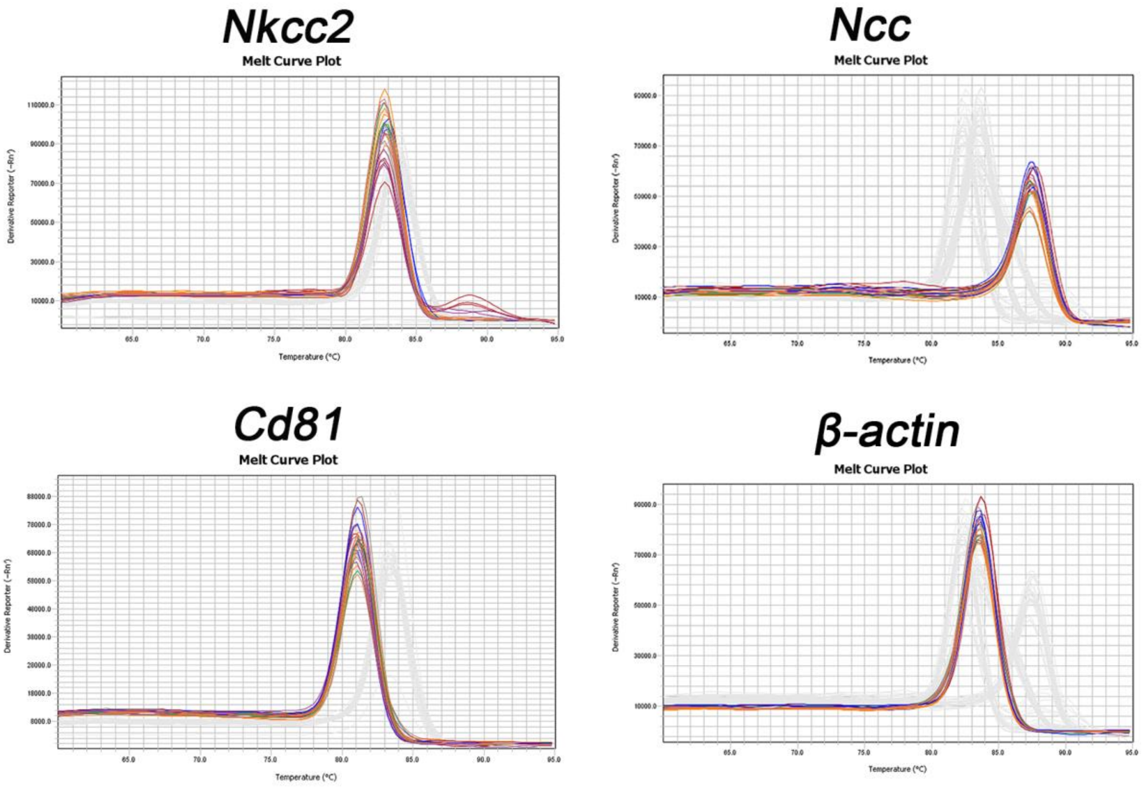

Figure S4. The QPCR product melting curves for Nkcc2, Ncc, Cd81 and β-actin. Each gene product against corresponding primers exhibited a single peak within a reasonable breakdown temperature range, indicating that the amplified double-stranded DNAs were single discrete species.

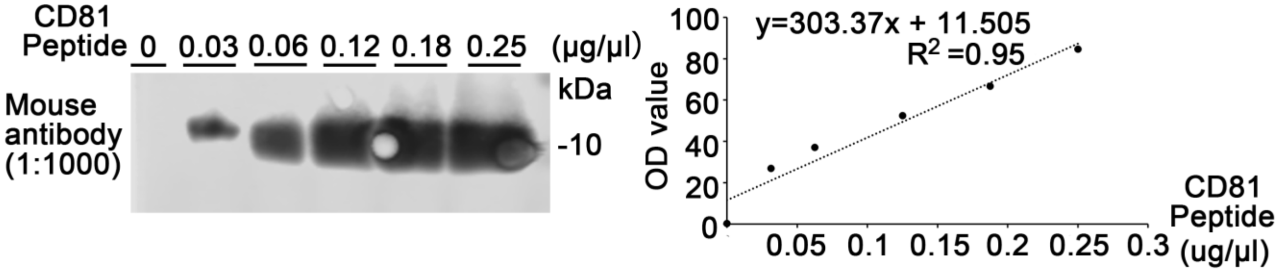

Figure S5. Standard curve of CD81 peptide detected by the mouse monoclonal antibody. The loading concentrations of CD81 peptide were 0, 0.03, 0.06, 0.12, 0.18, 0.25 μg/μl respectively. The CD81 antibody detected the bands around 10kDa and matched the expected size for the peptide with 99 amino-acid residuals. The OD values against the peptide concentrations presented a y=303.37x + 11.505 equation with R^2=0.95.

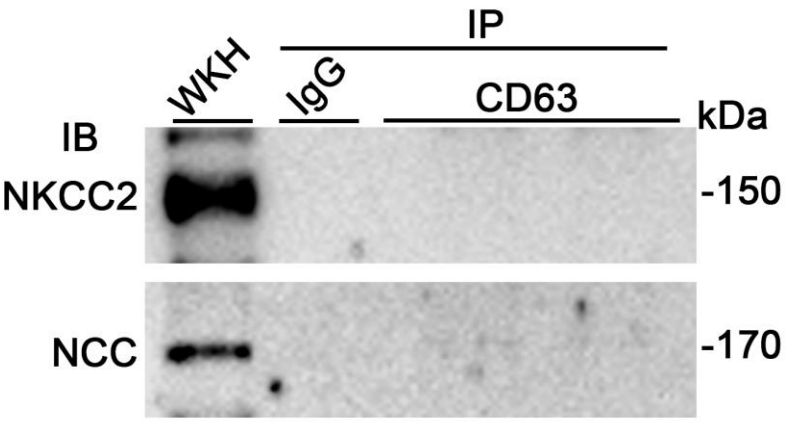

Figure S6. Co-immunoprecipitation of CD63 with NKCC2 or NCC from rat whole kidney homogenates (WKH). NKCC2 (rabbit polyclonal antibody) or NCC (rabbit polyclonal antibody) was not detected in the immunoprecipitation (IP) complex pulled down with antibody against CD63 (mouse monoclonal) using mouse IgG as a negative control (no band) and WKH input as a positive control (bands around 140-160 kDa). Blots were repeated in 3 different rats.

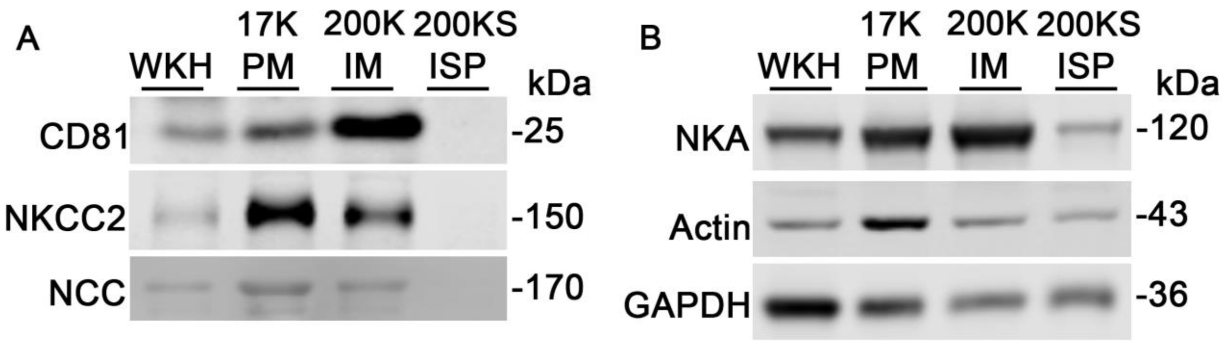

Figure S7. Protein abundances of CD81, NKCC2 and NCC in different membrane fractions (A) and characterization of renal membrane fractions prepared by differential centrifugation (B). 40 μg of protein samples were loaded from each fraction. Blots were repeated in 3 different rats. 17K: 17,000g centrifugation pellets. 200K: 200,000g ultracentrifugation pellets. 200KS:200,000g ultracentrifugation supernatants. WKH: whole kidney homogenates. PM: plasma membrane. IM: intracellular membrane. ISP: intracellular soluble protein.

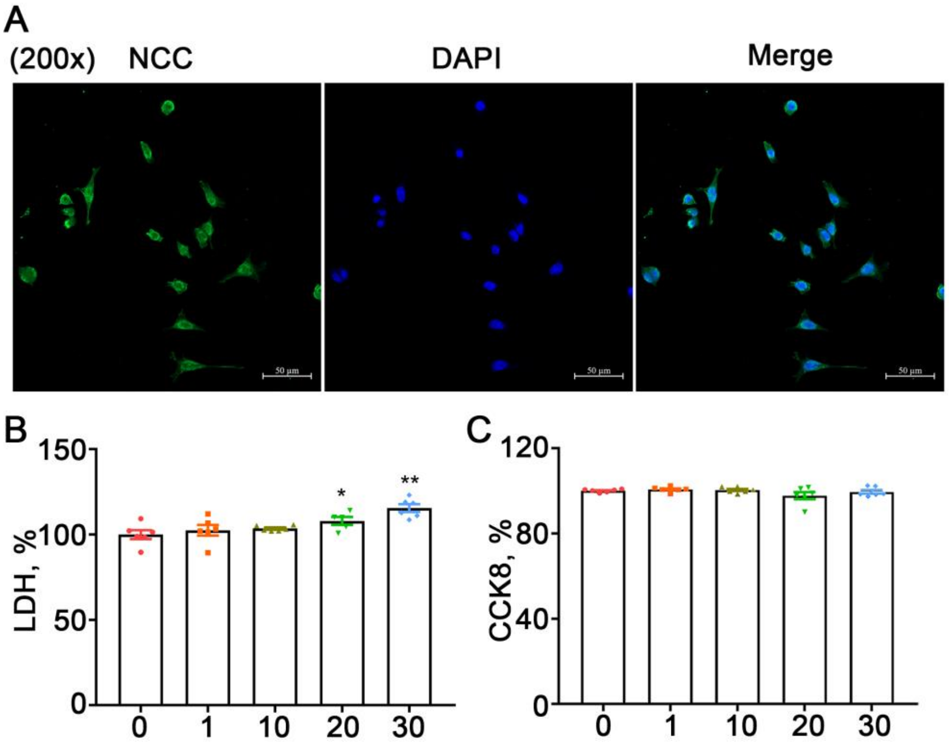

Figure S8. The purity, toxicity (B) and viability (C) of the mDCT cells. A. The immunofluorescent staining of the cells under confocal microscope (200x). NCC protein (green) was expressed in the plasma membranes and sub-cellular areas of the all cells viewed. The nuclei were stained with DAPI (blue). More than 10 fields were viewed; B. The LDH release rates of the cells treated with different concentrations of LPS for 24 hr. They were similar at all concentrations below 20 μg/mL; C. The CCK8 growth rates of the cells treated with different concentrations of LPS for 24 hr. Data were shown as mean ±SE, n=6/group, *P<0.05, **P<0.01 vs vehicle, one-way Analysis of Variance, Holm-Sidak Method.

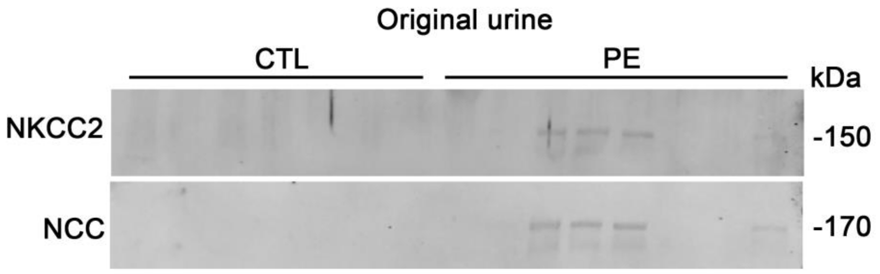

Figure S9. Immunoblots of NKCC2 and NCC in original urine samples from PE patients and controls. Loading volumes of the urine samples were normalized by urine creatinine. For NCC, 1 out of 16 (6.25%) in controls was positive while 10 out of 20 in PE were positive (50%). For NKCC2, all controls were negative while 8 out of 20 in PE were positive (40%).

Table S1. The information of the primers for Q-PCR

Name	Sequence (5' to 3')	Catalog number	Expected amplicon size	Gene ID
Nkcc2-F	TTCGGTGGGTCAATAGGCTT	NJP2019060005055	193bp	25065
Nkcc2-R	TCCCAGCTACTGAGATCCCT	NJP2019060005056		
Ncc-F	TACCCGCCTACGAACACTAC	NJP2019060005051	98bp	54300
Ncc-R	TTCCTGCTTGAGGAACGAGT	NJP2019060005052		
Cd81-F	AGATGATCCTGAGCATG	NJP2019080019014	101bp	25621
Cd81-R	GGTAACAGGAAAGTTCAGAAC	NJP2019080019015		
β-actin-F	CACCATGTACCCAGGCATTG	NJP2019060005057	173bp	296654
β-actin-R	CCTGCTTGCTGATCCACATC	NJP2019060005058		

Table S2. Metabolic cage data for Sprague–Dawley rats with LPS-PE on GD18

Group (n)	Vehicle (5)	LPS (6)
Body weight (g) on GD5	273±3	272±2
Body weight (g) on GD18	334±6	336±8
Urine (ml/day)	19.4.±2.3	17.1±1.8
Urine potassium (mmol/day)	3.1±0.9	2.2±0.5
Urine sodium (mmol/day)	0.5±0.1	2±0.6*
Urine chloride (mmol/day)	1.12±0.03	3.42±1.3*
Serum potassium (mmol/L)	5.2±0.4	5.2±0.8
Serum sodium (mmol/L)	142.7±3.8	127.7±2.4*
Serum chloride (mmol/L)	98.2±3.2	88.3±1.6*
Creatinine clearance (ml/min)	1.4±0.2	2.3±0.6

Urines and sera were collected on GD18. Data are shown as mean ± standard error, n=number of rats, student t-test, *$p<0.05$.

Table S3. General information of PE patients and controls.

Group (n)	CTL(16)	PE(20)
Age (years)	27 ±1	31 ±1*
Body weight (kg)	77 ±2	78 ±3
Height (cm)	161 ±1	161 ±1
Heart rate (beats/min)	82 ±3	89 ±3
Respiration rate (times/min)	19.5 ±0.2	19.3 ±0.2
Gravidity	1.7 ±0.3	2.4 ±0.4
Parity	1.3 ±0.1	1.6 ±0.2

The clinical information was recorded when PE was diagnosed and urine samples were collected.

Data are mean ± standard error, n= number of subjects. *p<0.05, student t-test.

Table S4. Biochemistry measurement of PE patients and controls

Group	CTL (n)	PE (n)
Urine potassium (mmol/mg of Cr)	0.05±0.01(16)	0.04±0.01(20)
Urine sodium (mmol/mg of Cr)	0.14±0.02	0.17±0.03
Urine chloride (mmol/mg of Cr)	0.14±0.02	0.14±0.02
Urine Creatinine (mmol/L）	8±1.1	8.4±1.1
Serum potassium (mmol/L)	4.6±0.7(9)	4.1±0.1(12)
Serum sodium (mmol/L)	137.6±0.9	138.3±0.5
Serum chloride (mmol/L)	105.1±0.9	103.2±0.4
Serum creatinine (μmol/L)	55.3±4.1	61.2±7.2

The clinical information was recorded when PE was diagnosed and urine samples were collected. Data are shown as mean ±standard error, n=number of subjects, student t-test.
